# Isolation and characterization of *Toxoplasma gondii* from captive caracals (*Caracal caracal*)

**DOI:** 10.1016/j.ijppaw.2020.10.006

**Published:** 2020-10-23

**Authors:** Nan Jiang, Shilin Xin, Junbao Li, Chunlei Su, Longxian Zhang, Yurong Yang

**Affiliations:** a College of Veterinary Medicine, Henan Agricultural University, Zhengzhou, PR China; b Zhengzhou Zoo, Zhengzhou, PR China; c Department of Microbiology, University of Tennessee, Knoxville, USA

**Keywords:** *Toxoplasma gondii*, Caracal (*Caracal caracal*), Isolation, Intermediate host, Genotyping, Virulence, China

## Abstract

*Toxoplasma gondii* infects most warm-blooded animals, including humans. Felids can serve as both intermediate and definitive hosts for *T. gondii*. However, there is no direct evidence to prove the caracal (*Caracal caracal*) is an intermediate host for *T. gondii*. Here, we report *T. gondii* infection in two caracals in a zoo from China. Antibodies against *T. gondii* were found in both caracals by modified agglutination test (MAT) (cut-off titer: 1:25). Tissue cysts were observed in the leg and tongue muscles of caracal case^#^ 1. These cysts were confirmed as *T. gondii* by immunohistochemical staining and *T. gondii* was detected by polymerase chain reaction (PCR). Viable *T. gondii* strain was isolated from the striated muscles of caracal case^#^ 2 and designated as TgCaracalCHn1. DNA from tachyzoites obtained from cell cultures was characterized by RFLP-PCR using ten markers (*SAG1, SAG3, SAG2, BTUB, c22-8, GRA6, c29-2, PK1, L358,* and *Apico*) and the virulence genes (*ROP5* and *ROP18*). The results indicate that this isolate belongs to ToxoDB genotye #2 (Type III). The virulence of this isolate was evaluated in BALB/c mice. A dose of 10^4^ TgCaracalCHn1 tachyzoites was non-lethal to mice. Tissue cysts were found in brain tissues of infected mice. This result confirmed that the TgCaracalCHn1 is non-virulent to mice. Current study documents first isolation of viable *T. gondii* strain from caracal and also indicates that caracal can act as new intermediate host for *T. gondii*.

## Highlights

• *T. gondii* infection in caracals was detected and confirmed by serological and molecular analysis, immunohistochemistry, and bioassay in mice.
• One viable *T. gondii* strain was isolated from caracal and designated as TgCaracalCHn1, ToxoDB#2.
• This report provides direct evidence of caracal as an intermediate host of *T. gondii.*

## Graphical abstract

**Figure:**
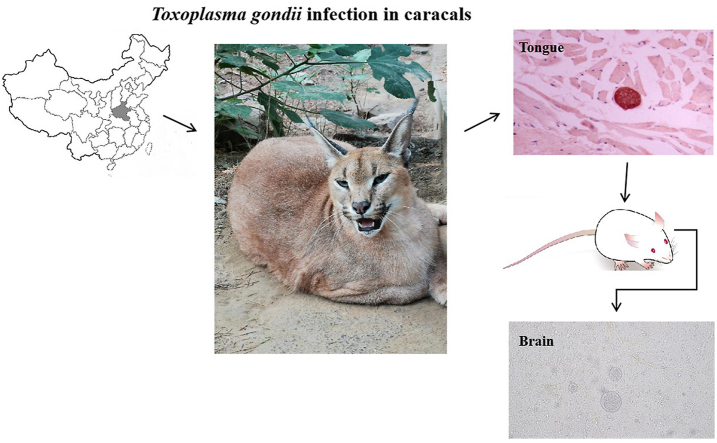

## 1 Introduction

*Toxoplasma gondii* is an intracellular parasite that could infect most mammals and birds, including humans (Dubey, 2010). A quarter of the world's population is infected with this parasite (Pappas et al., 2009; Waldman et al., 2020). Felids, only known definitive host of *T. gondii*, shed unsporulated oocysts in their feces and contaminate environment (Dubey, 2009). Furthermore, cats infected with *T. gondii* for the first time excrete the oocysts, and when antibodies in the body gradually decrease, they can be infected again and re-shed oocysts (Dubey, 1976, 1995; Zulpo et al., 2018). Under warmer and moister environmental conditions, the matured oocysts can remain infective for more than one year (Dubey, 2010). The *T. gondii* infected felids in zoos could be potential contamination source for environment, other animals and tourists.

The caracal (*Caracal caracal*) is a medium-sized cat native to Asia and Africa. The wild caracals prey on small mammals, birds, and rodents (Livingston, 2009). Chickens, rabbits, raw pork and beef are the main food for captive caracals. Currently, only some studies reported the presence of seroconversion antibody to *T. gondii* in caracals (de Camps et al., 2008; Spencer et al., 2003; Gomez-Rios et al., 2019; Serieys et al., 2019; Seltmann et al., 2020; Dubey et al., 2010). Varied rate of *T. gondii* infection has been documented in caracals around the world (Table 1). However, there is no report published on viable *T. gondii* strain isolated from this animal yet. In this study, we investigated *T. gondii* infection in two captive caracals from a zoo in China and demonstrated isolation of a strain from the striated muscles using mice bioassays.

**Table 1:** Reports of *Toxoplasma gondii* infection in caracals (Caracal caracal) around the world.

Region/Country	No. positive/No. tested	% Positive	Test	Antibody titers	References
United States	2/4	50.0	MAT	1:400 ≧1:3200	de Camps et al. (2008)
United States	1/1	100.0	IFAT	1:100	Spencer et al. (2003)
Mexico	1/1 1/1	100.0	ELISA IFAT	– 1:160	Gomez-Rios et al. (2019)
South Africa	24/29	82.8	IFAT	–	Serieys et al. (2019)
Namibia	10/15	66.7	ELISA	–	Seltmann et al. (2020)
United Arab Emirates	6/7	85.7	MAT	1:100 (2), 1:200 (3), 1:3200 (1)	Dubey et al. (2010)
China	0/1	–	ELISA	–	Zhang et al. (2001)
China	1/1	100.0	DAT	≧1:16	Zhang et al. (1991)
China	1/1	100.0	DAT	1:16	Shi et al. (1992)
China	2/2	100.0	MAT	1:800 ≧1:200	This study
Total	49/63	77.8			

-: Unknown.

IFAT = Indirect fluorescent antibody.

ELISA = Enzyme-linked immunosorbent assay test.

MAT = Modified agglutination test.

DAT = Direct agglutination test.

## 2 Materials and methods

### 2.1 Samples collection and sites

Two captive caracals died of a respiratory disorder in a zoo (34°46′ N, 113°39′ E, Henan) from China in 2017–18 (Table 2). Fresh kidney, heart, spleen, lung, liver, lymphonodus, tongue, brain, diaphragm, and leg muscle samples from caracals were submitted to the Laboratory of Veterinary Pathology of Henan Agricultural University (Zhengzhou, Henan, China) for pathological diagnosis; *T. gondii* infection in caracals were investigated.

**Table 2:** Clinical symptoms and isolation of *Toxoplasma gondii* in caracals from the current study.

Animal ID	Received Date	Sex, age	Clinical signs	Pathological findings	MAT titer	Cysts by HE	Cysts by IHC	PCR^a^	Mice bioassay^b^	
									BALB/c	γ-IFN^−/−^
Case^#^1	June 12, 2017	Male, Adult	Dry nose, depressed, anorexia.	Pulmonary edema, interstitial phrenitis.	1:800	+ Tongue, leg muscle	+	+	0/5, 0/5, 0/2	nd
Case^#^2 **(TgCaracalCHn1)**	March 20, 2018	Female, Adult	Aggressive behavior toward other caracals, hematemesis, and died.	Suppurative pneumonia, bacteremia.	≧1:200^c^	–	nd	–	0/4, 1/5 (from KO mouse), 1/5, 3/3, 5/5	1/1

nd: not done.

a Primer were TOX5/TOX8.

b Number of positive mice/number of inoculated mice.

c End titration not performed.

### 2.2 Serological examination by modified agglutination test (MAT)

Heart fluid (0.5 mL) from caracals was collected directly. Antibodies against *T. gondii* were detected in the heart fluid by MAT (Dubey and Desmonts, 1987). *Toxoplasma gondii* antigen was obtained from the University of Tennessee Research Foundation (Knoxville, TN, USA; https://utrf.tennessee.edu/). Heart fluid was diluted two folds, starting from 1:25 till 1:12,800. Blank control (only reagents, no serum), positive and negative controls (sera from mice with and without *T. gondii* infection, respectively) were included in each plate.

### 2.3 Histopathological analysis

Collected tissues were fixed in 10% neutral buffered formalin. They were paraffin sectioned, then sections (5 μm thick) were stained with hematoxylin and eosin (H&E) routinely. Sections suspected for the presence of tissue cysts were stained with immunohistochemistry (IHC) (Su et al., 2019). The primary antibody used was rabbit anti-*T. gondii* polyclonal antibody. Anti-rabbit IgG was used as secondary antibody (product code: ab64264, Abcam, Cambridge, MA, USA). Brain sections of VEG *T. gondii*-infected mouse (provided by JP Dubey, ARS, USDA) were used as positive controls.

### 2.4 Isolation of *T. gondii* from caracal muscle by bioassay in mice

Tissues (50-g, including heart, tongue, leg muscle, and diaphragm) from two caracals were homogenized and digested in pepsin solution, respectively (Dubey, 2010). The homogenates were inoculated into BALB/c mice (n = 4–5) and/or gamma interferon (γ-IFN) knockout mice (n = 1) subcutaneously. Specific pathogen-free BALB/c mice were provided by Laboratory Animal Center of Zhengzhou University (Zhengzhou, China). IFN-γ^−/−^ mice were supplied by Jackson Laboratory (product code: 002287). After inoculation, tissue (lung, mesenteric lymph nodes or brain) smears of dead mice were examined for *T. gondii* parasites. Survivors were bled on day 60 post-inoculation (DPI), and serum from each mouse was tested for *T. gondii* antibodies by MAT with 1:25 and 1:200 dilution. If parasites were not found in the lung, mesenteric lymph nodes or brain of mice, the tissues (brain, heart, lung, mesenteric lymph nodes, tongue) of mice were ground and subcutaneously passage into new groups of mice (n = 2–4).

### 2.5 Detection of *T. gondii* DNA by PCR

DNA was extracted from the pepsin digested juice (striated muscles) using DNA extraction kit (Tiangen Biotec Company, DP304, China). *Toxoplasma gondii* DNA was amplified by PCR using the primer pair TOX5-TOX8. The expected products for *T. gondii* were 450 bp in length (Reischl et al., 2003). Positive and negative controls [DNA extracted from brain of mice infected with *T. gondii* (VEG strain) and not infected, respectively] were included in each batch.

### 2.6 *In vitro* cultivation and genetic characterization

Tissue homogenates (brain for chronic infection, lung and mesenteric lymph nodes for acute infection) from *T. gondii-*positive mice were seeded into Vero cells (Dubey, 2010). Cell cultured tachyzoites were collected. DNA was extracted from tachyzoites by DNA extraction kit (No. DP304, Tiangen Biotec Company, China). The *T. gondii* strain genotyping was performed by PCR-RFLP using ten genetic markers *SAG1, SAG2* (5′-and 3′-*SAG2*, alt. *SAG2*), *SAG3, GRA6, BTUB, L358, PK1, c22-8*, *c29-2,* and *Apico* (Su et al., 2010).

The virulence protein gene allele types of *ROP5* and *ROP18* were measured as previously reported (Shwab et al., 2016a, 2016b). Briefly, the upstream promoter insertion sequence (UPS) and a repetitive sequence (DEL) of *ROP18* was amplified by PCR using the external primers (ROP18-DelFext: CTCGTCGACCACACAGCTAA; ROP18-UPSRext: GA

GTGCTTTCTGTCGCTCCT; ROP18-UPSFext: TTTTATCGACATCCCGCTTC; ROP18-UPSRext: GAGTGCTTTCTGTCGCTCCT) and internal primers (ROP18-DelFint: AGTTCCCTTCCCTGGTGTCT; ROP18-DelRint2: CACCGCAAGACAGGCTGTCTTC; ROP18-UPSFint: CACAGCATGAGCTTAAGAGTTG; ROP18-UPSRint2: ACAAACTGGACTGGGGTGAG). The DEL sequence was double digested with restriction enzymes *Scr*FI and *Mfe*I to distinguish alleles 1, 2 and 4. Type III allele had positive UPS PCR products. *ROP5* was amplified by nested PCR using the external primers (ROP5-Fext: GGACAGACGCAGGCT TTTAC; ROP5-Rext: TCAAACGTCCTGACACTTCG) and internal primers (ROP5-Fint: TGTGGCAGTTCAGTCTCAGC; ROP5-Rint: TCGAAGTTGAGGAACCGTCT). The ROP5 PCR products were digested with restriction enzymes *Fsp*BI to distinguish alleles 1, 2, 3 and 4. Restriction enzymes *Bst*UI was used to distinguish alleles 5 and 6. Respective controls of *T. gondii* DNA were included in each batch.

### 2.7 Virulence assessment of *T. gondii* isolated from caracal in mice

The virulence of *T. gondii* isolated from caracal was evaluated in BALB/c mice (Dubey et al., 2012; Saraf et al., 2017). *Toxoplasma gondii* tachyzoites were counted in hemocytometer, and diluted 10-fold from 10^−1^ to10^−8^ to reach an endpoint of less than 1 tachyzoite. Tachyzoites (<1, 10^0^, 10^1^, 10^2^, 10^3^, and 10^4^) were inoculated into five mice for each dilution, intraperitoneally. The clinical symptoms were observed daily. At 60 DPI, the surviving mice were bled and tested for antibodies against *T. gondii* by MAT with titers 1:25 and 1:200. The mice were euthanized at 61 DPI. *Toxoplasma gondii* cysts were checked and the number of cysts in mouse brains was recorded (Dubey et al., 2012). Either presence of parasite or positive on serology, the mice were considered infected *T. gondii.*

### 2.8 Ethics

This study was approved by the Beijing Association for Science and Technology (SYXK [Beijing] 2007-0023) and the Institutional Animal Use Protocol Committee of the Henan Agricultural University, China.

### 2.9 Statistical analysis

Statistical analysis was performed by the GraphPad Prism 6.0 (GraphPad Software Inc., San Diego, CA, USA). Data were analyzed using the chi-squared test. A *P* < 0.05 was considered significant.

## 3 Results

### 3.1 Clinical symptoms and pathologic lesions

**Fig. 1:**
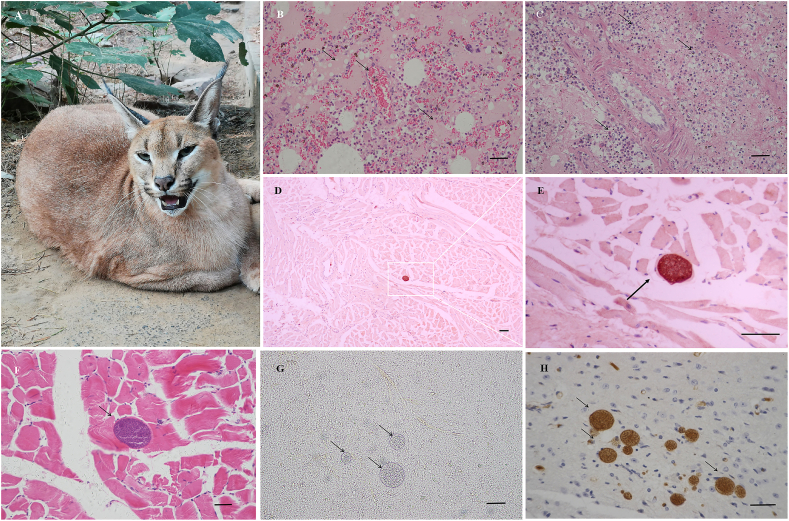
Histopathological findings in the caracals (*Caracal caracal*). A. A caracal from the zoo (China); B. Pulmonary edema, case ^#^1, lung, caracal, H&E; C. Suppurative pneumonia, case ^#^2, lung, caracal, H&E; D. *Toxoplasma gondii* cyst in the skeletal muscle cell, case#1, tongue, caracal, IHC; E. Magnified Fig. 1 D, case ^#^1, tongue, caracal, IHC; F. *Toxoplasma gondii* cysts in the skeletal muscle cell, case ^#^1, leg muscle, caracal, H&E; G. *Toxoplasma gondii* TgCaracalCHn1 cysts in brain, mice, 51 DPI, squashed section, unstained; H. *Toxoplasma gondii* TgCaracalCHn1 cysts in the brain, 10^4^ tachyzoites, 61 DPI, mice, IHC; Bar = 50 μm.

Two caracals from a zoo were submitted for pathological diagnosis (Table 2, Fig. 1A). Caracal case^#^ 1, a male adult, presented signs of dry nose, depression, anorexia, and eventually died in a few days. Grossly, it showed pulmonary congestion, hepatomegaly, enlarged kidney, and spleen atrophy. Microscopically, hepatocyte vacuolar degeneration, severe pulmonary edema caused by bacterial infection was observed (Fig. 1B). Pulmonary edema was the leading cause of respiratory failure and death. Oval-shaped *T. gondii*-like cysts were found in the striated muscle cells of leg and tongue (Fig. 1D–F). Strong immune staining for *T. gondii* in these cysts was observed by IHC (Fig. 1D and E). The sizes of these cysts ranged between 30–45 μm × 26–39 μm in H&E sections. No signs of inflammation were found around the cysts.

Caracal case^#^ 2 was a female adult that had a history of fight with other caracals, had hematemesis and died. On gross examination, enlarged pale liver, soft enlarged spleen, multiple consolidation and congestions in lung were observed. Histopathological findings were suppurative pneumonia (Fig. 1 C) and acute necrosis splenitis. Both of the caracals did not die of acute toxoplasmosis.

### 3.2 Serological examination and DNA detection

Antibodies against *T. gondii* were detected in both caracals with MAT titers higher than 1:200. DNA of *T. gondii* was detected in one caracal (case ^#^1; Table 2).

### 3.3 Isolation of *T. gondii* in mice and cell cultures

The striated muscles of two caracals were bioassayed in mice individually (Table 3).

**Table 3:** Isolation of *T. gondii* from caracal muscles by bioassay in mice.

Group	Inoculated Date	Sample	Mice ear tag number	Mice species	Results	Subpassaged
Tox^#^ 24-1	20170615	Caracal^#^ 1 tissues	^#^2411,^#^2412,^#^2413,^#^2414,^#^2415 DXM^a^	Wild type	^#^2414 Died at 46 DPI^b^, brain negative other four mice IgG negative, brain negative at 60 DPI	M^#^2414 for Tox^#^ 24-3
Tox^#^ 24-2	20170615	Caracal^#^ 1 tissues	^#^2416,^#^2417,^#^2418,^#^2419,^#^2420	Wild type	All mice IgG negative, brain negative at 60 DPI	Discard
Tox^#^ 24-3	20170801	Tox^#^ 24–1, M^#^2414 tissues	^#^2462,^#^2463	Wild type	All mice IgG negative, brain negative at 50 DPI	Discard
Tox^#^ 24-4	20180323	Caracal^#^ 2 tissues	^**#**^**153**	IFN-γ^−/−^	Died at 14 DPI, lung, mesenteric lymph nodes negative	M^#^153 for Tox^#^ 24-5
			^#^547,^#^576,^#^471,^#^473 DXM	Wild type	^#^547,^#^576,^#^471 IgG negative, brain negative at 30 DPI ^#^473 IgG 1:25, brain negative at 280 DPI	
Tox^#^ 24-5	20180406	Tox^#^ 24-4, **M**^**#**^**153** tissues	^**#**^**358**,^#^404,^#^346,^#^384,^#^406	Wild type	^#^358 died at 37 DPI, IgG positive, but lung and brain negative other four mice IgG negative, brain negative at 50 DPI	M^#^ 358 for Tox^#^ 24-6
Tox^#^ 24-6	20180514	Tox^#^ 24-5, **M**^**#**^**358** tissues	^#^668,^#^670,^#^682,^#^684, ^**#**^**821**	Wild type	^#^670, IgG negative, brain negative at 67 DPI ^#^821, IgG positive at 30 DPI, brain negative at 234 DPI ^#^682,^#^684,^#^668, IgG negative at 30 DPI, brain negative at 270 DPI	M^#^ 670 for Tox^#^ 24-7; M^#^ 821 for Tox^#^ 24-8
Tox^#^ 24-7	20180720	Tox^#^ 24–6, M^#^670 tissues	^#^808	IFN-γ^−/−^	All mice IgG negative, brain negative at 50 DPI	Discard
			^#^812,^#^686,^#^9926	Wild type		
Tox^#^ 24-8	20190208	Tox^#^ 24-6, **M**^**#**^**821** tissues	^**#**^**883,**^**#**^**884,**^**#**^**885**	Wild type	All mice IgG positive, brain positive at 12-270 DPI	M^#^ 885 for Tox^#^ 24-9
Tox^#^ 24-9	20190220	Tox^#^ 24-8, **M**^**#**^**885** tissues	^**#**^**886,**^**#**^**887,**^**#**^**888,**^**#**^**889,**^**#**^**890**	Wild type	All mice IgG positive at 30 DPI	Save

Bold letters marked *Toxoplasma gondii* positive mice.

a DXM, Treatment dexamethasone phosphate (10 μg/mL) for 3 days in drinking water.

b DPI, days post inoculation.

For the Tox^#^ 24-1 and Tox^#^ 24-2 group (case^#^ 1), none of the mice (n = 5, respectively) had antibodies for *T. gondii,* and no bradyzoite was observed at 60 DPI*.*

For the Tox^#^ 24-4 group (case^#^ 2), IFN-γ^−/−^ mouse (M^#^ 153) was died at 14 DPI, but no tachyzoite was observed in the lung and mesenteric lymph nodes. The tissues (brain, heart, tongue, lung, and mesenteric lymph nodes) of mouse^#^ 153 were ground, and subcutaneously passaged to the Tox^#^ 24-5 group, 1 of 4 mice (M^#^358) was dead at 37 DPI, yet no parasites were observed in the lung and brain, then tissues from this mouse was subpassaged as described to Tox^#^ 24-6 mice. For the Tox^#^ 24-6 group, 1 of 4 mice (M^#^ 821) had seroconverted antibodies for *T. gondii*, but no cyst was found in the brain at 234 DPI; this mouse was subpassaged to the Tox^#^ 24-8 group. Sera of all (3/3) of the mice serum tested positive for anti-*T. gondii* antibody at 30 DPI, and eight cysts in whole brain were detected in mouse^#^ 884 at 270 DPI. This isolate from the mouse brain was propagated in cell culture successfully (12 DPI) and designated as TgCaracalCHn1. Isolated strain of *T. gondii* was identified as ToxoDB#2 (type III) based on ten genetic makers. The *ROP18* and *ROP5* gene allele type of this isolate was 3/3.

### 3.4 Virulence evaluation of *T. gondii* TgCaracalCHn1

After the inoculation of mice with TgCaracalCHn1 tachyzoites, till 60 DPI, the positive mice had no symptoms. 10^3^ tachyzoites of *T. gondii* infected 100% (5/5) of mice. *Toxoplasma gondii* cysts were detected in mouse brains (Fig. 1 G and H). The numbers were from 170 to 5300 cysts per mouse brain. The cysts number in mouse brains was not increased significantly with higher doses of tachyzoites (*P* > 0.05) (Table 4).

**Table 4:** Evaluation of the virulence of *Toxoplasma gondii* TgCaracalCHn1 strain in BALB/c mice.

No. of tachyzoites	No. of infection/No. of inoculation (%)	Days of survival/number of mice	No. of brain cysts
10^4^	5/5 (100%)	≥60DPI/5	292.5 ± 107.6
10^3^	5/5 (100%)	≥60DPI/5	170.0 ± 89.4
10^2^	3/5 (60%)	≥60DPI/5	236.7 ± 87.6
10^1^	2/5 (40%)	≥60DPI/5	570.0 ± 70.0
1	1/5 (20%)	≥60DPI/5	5300
<1	0/5 (−)	≥60DPI/5	Not found
Blank control	0	≥60DPI/5	Not found

## 4 Discussion

To the best of our knowledge, this is the first report of isolated viable *T. gondii* from caracal. This is also the first study to document *T. gondii* cysts directly from the striated muscles of caracals; however earlier studies only detected the antibodies to *T. gondii* in caracals (Serieys et al., 2019; de Camps et al., 2008; Zhang et al., 1991, 2001; Shi et al., 1992; Spencer et al., 2003; Gomez-Rios et al., 2019; Seltmann et al., 2020; Dubey et al., 2010). In this study, isolation *T. gondii* stain from caracal case ^#^ 1 was unsuccessful. The low cyst load and avirulence of *T. gondii* may be relevant to the isolation result.

Genotype of the isolate TgCaracalCHn1 was identified as ToxoDB#2 (type III). ToxoDB#2 is widely distributed worldwide, including Asia, Africa, South Europe, North America, South and Central America (Chaichan et al., 2017; Shwab et al., 2018; Halos et al., 2010; Dubey, 2010; Dubey and Crutchley, 2008; Dubey et al., 2013, 2014). ToxoDB#2 *T. gondii* strains have found in cats (Yang et al., 2015) and sheep (Jiang et al., 2020) from central China, indicating that except for ToxoDB#9, ToxoDB#2 is one of the major endemic genotype in China. The *ROP18*/*ROP5* genotype combination (3/3) suggests that this strain is avirulence for mice (Shwab et al., 2016a), which matched with the mouse virulence evaluation in this study.

The ingestion of bradyzoites is the most effective ways of transmission *T. gondii* in felids (Dubey, 2006). In this study, caracals were bred in zoo, and their diets were fresh raw beef, pork and mutton. They also eat birds, rodents and insects when available. The seroprevalence of *T. gondii* infection for swine, cattle, and sheep from China was 32.9%, 9.1% and 11.8%, respectively (Dong et al., 2018). A previous study showed feeding frozen tissues, keeping animals in enclosures using fences with small mesh sizes, and wearing gloves for breeder could decreased *T. gondii* infections in captive felids (Lücht et al., 2019). Furthermore, oocysts shed by cats may be another possible source of *T. gondii* infection for felids ( Dubey et al., 1996). The seroprevalence of *T. gondii* IgG antibodies was 88.9% (8/9) in captive felids (Yang et al., 2017), and 80.0% (8/10) in captive tigers (Yang et al., 2019) from central China. The *T. gondii* oocysts can be spread mechanically by earthworms, cockroaches, and flies. They can also be spread through shoes, or equipment from keepers to other members of the public (de Camps et al., 2008). All of this increases the risk of *T. gondii* infection in zoo animals, including caracals.

Pre-freeze meat to inactivate *T. gondii* tissue cysts may be necessary to prevent transmission of *T. gondii* in zoo felids. Meat frozen at −12 °C for seven days is a valid strategy to reduce *T. gondii* infection in caracals or other felids. Feces of caracals or other felids should be cleaned up daily to prevent sporulating oocysts.

## Availability of data and material

The datasets used and/or analyzed in the current study are available from the corresponding author upon reasonable request.

## Funding

This study was financed by the Key research projects of Henan higher education institutions (21A230009) and China Postdoctoral Science Foundation (2016M600577).

## Declaration of competing interest

The authors declare no competing interests. None of the authors of this report has financial or personal relationships with other people or organizations that could inappropriately influence its content.
